# Striving to maintain a dignified life for the patient in transition: Next of kin’s experiences during the transition process of an older person in transition from hospital to home

**DOI:** 10.3402/qhw.v10.26554

**Published:** 2015-03-05

**Authors:** Sigrun Hvalvik, Inger Å. Reierson

**Affiliations:** 1 Faculty of Health and Social Studies, Telemark University College, 3901 Porsgrunn, Norway; 2 Centre for Caring Research-Southern Norway, Telemark University College, 3901 Porsgrunn, Norway and University of Agder, 4898 Grimstad, Norway

**Keywords:** Relatives, adult, alteration, hospital care, home care, phenomenological hermeneutic study

## Abstract

Next of kin represent significant resources in the care for older patients. The aim of this study was to describe and illuminate the meaning of the next of kin’s experiences during the transition of an older person with continuing care needs from hospital to home. The study has a phenomenological hermeneutic design. Individual, narrative interviews were conducted, and the data analysis was conducted in accordance with Lindseth and Norberg’s phenomenological hermeneutic method. Two themes and four subthemes were identified and formulated. The first theme: “Balancing vulnerability and strength,” encompassed the subthemes “enduring emotional stress” and “striving to maintain security and continuity.” The second theme: “Coping with an altered everyday life,” encompassed “dealing with changes” and “being in readiness.” Our findings suggest that the next of kin in striving to maintain continuity and safety in the older person’s transition process are both vulnerable individuals and significant agents. Thus, it is urgent that health care providers accommodate both their vulnerability and their abilities to act, and thereby make them feel valued as respected agents and human beings in the transition process.

Since the Coordination Reform (Report No 47 to the Storting, 2009) took effect in January 2012, the number of older patients discharged from the hospital to their homes and with further need of professional help has increased in Norway. Accordingly, frail older patients return to their homes coping with multiple chronic conditions and complex medication regimens. They are more dependent on care and have a higher risk for readmission than before the reform was introduced (Heggestad, 2002; Norwegian Knowledge Centre for the Health Services, 2012). This could also be a challenging situation for the next of kin. The Norwegian Ministry of Health and Care Services has released several reports in recent years: Report No. 47 (2008–2009) to the Storting, Report No. 29 (2012–2013), and Report No. 10 (2012–2013). These reports have a strong focus on improving the quality of the health care services and have also taken into account that the next of kin is a valuable resource in health care situations. The reports, however, also point out that being the next of kin could be so stressful that they themselves might be in need of help. The way the next of kin are included in the transition process might therefore be of great importance in multiple ways. To involve them in a proper and respectful manner will probably increase next of kin’s ability to support the patient, and at the same time prevent the occurrence of stress and strain in the transition process.

Several problems may arise in the transfer from one care setting to another, such as lack of information, errors related to medications, and unfamiliarity with the patients (Boling, 2009; Coleman & Berenson, 2004; Golden, Tewary, Dang, & Roos, 2010). These problems do not only contribute to the rates of readmission and increase of economic costs but also affect the patients’ lives, and the lives of the next of kin. In addition, the patients often face challenges related to illness and health failure, which frequently involve loss and changes that are undesirable (Schumacher, Jones, & Meleis, 1999). Such challenges will also affect the next of kin. During the transition process, the patients are particularly vulnerable and need to develop new coping strategies, new relationships, and new skills for dealing with life (Meleis & Trangenstein, 1994). Simultaneously, it is an ambition that the patients are informed and supported in ways that enable them to participate actively in making necessary health care decisions. Also, the right to determine their own aims should be respected (Norwegian Ministry of Health and Care Services presents in Report No. 10 [2012–2013] to the Storting). To succeed in achieving these goals, the contribution of the next of kin may be crucial.

Nolan, Davies, Brown, Keady, and Nolan (2004) claim that acknowledging the pivotal role of the family in the caring situation is crucial. They further argue that all parties involved in the caring situation should experience a relationship that promotes a sense of security, belonging, continuity, purpose, achievement, and significance. In this context, they suggest a framework based on the idea of relationship-centred care. The person-centred model, although promoting a more whole-person approach, is in their opinion not inclusive enough to embrace all the dimensions in the caring situation. McCormack and McCance (2010), however, argue that the idea of person-centred care like relationship-centred care encompasses all those involved in the caring action. In both frameworks, respect for values, and the importance of developing a relationship built on mutual trust and understanding is central (McCormack & McCance, 2010; Nolan et al., 2004). Furthermore, this is in accordance with the framework developed by Galvin and Todres (2013) which focused on humanization of care. Crucial in this framework is the very basic question: What makes people feel more human in situations in which they receive care? This question is also relevant and important in relation to the next of kin in the context of this study.

Studies related to the next of kin’s experiences during their older family member’s transitions from hospital to home seem to be sparse, nationally as well as internationally, after the year 2000. Some studies concerning the experiences of patients and their caregivers are identified by Naylor (2012) in an overview of transitional care of older adults, executed in the USA in the 1990s. These studies showed that, in general, a high proportion of elders and their caregivers reported substantial unmet needs, such as the need for information. The studies also demonstrated differences in expectations between patients, their families, and health care providers; and the need for increased patient and family involvement in decision making in the discharge process. Studies after 2000 have mainly focused on hospital discharge of older patients, readmission, and return to hospital (Bauer, Fitzgerald, Haesler, & Manfrin, 2009; Dedhia et al., 2009; Dunnion & Kelly, 2008; Golden et al., 2010). These studies pointed out communication and information as vital but insufficient between the health care providers, the patients, and the family caregivers. Other studies stress the role the family and significant others play in caring for the frail older patient, making care decisions, and acting on the patient’s behalf (Bull, Hansen, & Gross, 2000; Efraimsson, Sandman, Hyden, & Rasmussen, 2006).

Norwegian studies focusing on transitional care from hospital to home have mainly been conducted from a nursing perspective (Dale & Hvalvik, 2013; Danielsen & Fjær, 2010; Hvalvik & Dale, 2013; Olsen, 2013). In one of their studies, Hvalvik and Dale (2013) found that the nurses endeavoured to provide care based upon respect for the independent individual as a living whole. Their ambitions were, however, threatened by the caring context. In her PhD thesis, Olsen (2013) found that barriers associated with the nurse, interpersonal processes, and with the organization negatively influenced the information exchange between home care and hospital nurses, and could put the older patient in a vulnerable and exposed situation. There are also a number of studies related to the older persons’ transitions to nursing homes and the next of kin’s experiences (Bern-Klug, 2008; Eika, Søderhamn, Espnes, & Hvalvik, 2014; Lattimer, 2011). These studies, with somewhat different perspectives on the subject, indicate that new and additional research on the next of kin’s experiences of their old family member’s transition from hospital to home is required as well.

## Aim

The aim of this study was to describe and illuminate the meaning of the next of kin’s lived experiences during the transition of an older person with continuing care needs from hospital to home.

## Methods

### Design

To describe and illuminate the meaning of the next of kin’s lived experiences related to older family members’ transition from hospital to home, a phenomenological hermeneutic design was chosen. In both phenomenology and hermeneutic philosophy, the lifeworld perspective, focusing on how the world with its everyday phenomena is lived, experienced, acted, and described by humans, is fundamental (Dahlberg, Dahlberg, & Nystrøm, 2008). Leaning on this perspective means we believe that individuals and their existence can never be satisfactorily understood if they are not looked upon as living wholes. The same view can be associated with the value framework for the humanization of care developed by Galvin and Todres (2013). Galvin and Todres (2013) stress the importance of lifeworld-led care, where an understanding of the concrete, everyday experiences of people is used to underpin the care. Lifeworld-led care thus means that we need knowledge that understands both the freedoms and the vulnerabilities of peoples’ journeys as they struggle with different health-related conditions. To obtain knowledge of the essential meaning of lived experience, a phenomenological hermeneutical method, grounded in the philosophical assumptions of the French philosopher Paul Ricoeur, was used in this study. According to Ricoeur (1976) a text lives its own life in the sense that it should be objectified and separated from the narrator. The process includes a movement from understanding what the text says to what the text really talks about. In the process the researchers continuously move back and forth between the parts and the whole of the text. In this sense, the method provides a dialectic movement between understanding and explanation, and understanding and interpretation.

### Participants and setting

A purposive sampling method was used to recruit participants. In keeping with ethical considerations, contact with potential participants was established through the home care authorities. Health care professionals who were expected to recruit participants were informed about the study both verbally and in written form. They relayed information and descriptions of the study to the next of kin who fulfilled certain inclusion criteria. These criteria were as follows:

- Being the next of kin to an older patient (age 67 and older) who has made a transition from hospital to home in the previous 2–8 weeks and has further need of professional care
- Being a family member close to the patient, such as spouse, sons, or daughters
- Being cognitively able and having consent competence
- Being able to articulate experiences verbally

Those who gave a preliminary consent were contacted by the researchers by telephone to arrange a first meeting. In this meeting, the information was examined once more, and they were also reminded of their rights. If they still wanted to participate, they signed the informed consent form.

The participants consisted of one son, two spouses, and eight daughters. There were various reasons why the older persons had been hospitalized, such as acute disease or injury, or exacerbation of chronic disease. Some of them had received home care before hospitalization, whereas others started home care after returning home. A few were transferred from hospital to a municipal rehabilitation or short-term care facility before they came home with home care.

At least three factors were involved when the patients were transferred from hospital to home: the hospital, the purchaser unit, and the home care services.^1^ The hospital in this study had its own officer from the purchaser unit. This meant that another officer took over when the patient returned home.

### Data collection

The two researchers interviewed five and six participants, respectively. The purpose of the interviews was to obtain in-depth information about the next of kin’s experiences during the transition process and included their experiences with the hospital as well as with the home care services. Narrative interviews were considered convenient for this purpose. The participants were encouraged to tell as freely as possible about their experiences related to the older patient’s discharge from hospital, to the arrival at home. Furthermore, they were asked to tell about negative as well as positive experiences during the transition process, and to expand or explain their views. Both researchers were also educated nurses and had professional as well as personal experiences with the phenomenon under investigation. To be aware of and broaden their pre-understanding, they discussed and reflected upon the subject before and during the interview process. In this way, they endeavoured to move beyond their pre-understanding, and also to maintain an open and flexible attitude in the interview situation (Gadamer, 1989). The interviews lasted about 1 h. They were audio taped and transcribed verbatim by the researchers.

### Data analysis

The interview text was analysed using a method developed by Lindseth and Norberg (2004). The method is inspired by the theory of interpretation presented by Ricoeur (1976) and aims to reveal the meaning of lived experience. The method comprises the following three steps. Step 1, *naïve reading*, is the phase where the researchers read the text several times in order to grasp its meaning as a whole. Step 2, the *structural analyses*, are the methodological instance of interpretation which can be performed in several ways. Step 3, *comprehensive understanding*, is related to the process of interpreting the text as a whole and arriving at a comprehensive understanding. In this process, the themes and subthemes are reflected on in relation to the research question and the context of the study as well as to relevant literature with an as open mind as possible.

Following these steps, the researchers first read the text several times to understand it as a whole. They further formulated a naïve understanding of the interview texts. Step 2 was executed as a thematic structural analysis. To confirm and widen the initial understanding of what the text said, the text was divided into meaning units. Those were reflected on in relation to the naïve understanding. The meaning units were condensed to grip what the text talked about. The meaning units were compared and rewritten and subthemes and themes were identified and formulated. The final subthemes and themes were formed and compared with the naïve understanding for validation. Finally in Step 3, the two researchers reflected together on the text, the naïve understanding, and the themes in relation to the aim and the context of the study to formulate a comprehensive understanding. In this process, they interpreted out of their pre-understanding as these cannot be set aside and thus constitute an essential condition for the dialogue with the text (Gadamer, 1989; Lindseth & Norberg, 2004).

### Ethical considerations

The research was executed in accordance with the Helsinki declaration. Approval for the study was obtained from the Norwegian Social Science Data Services [15.11.2013/36159/2/KH]. Formal access to the field was made through the home care authorities. Leaders, health care professionals, and participants were informed verbally and in written form about the study. The participants were informed at least twice about the study and were also reminded about the assurance of confidentiality, the nature of voluntary participation, and the right to withdraw at any time, before they signed the consent form (Beauchamp & Childress, 2009). They were also informed that they could contact their home care nurse if they needed to talk more after the interviews were finished.

## Findings

### Naïve reading

Being the next of kin to a frail older person in transition from hospital to home is challenging in several ways. Experiencing lack of vital information and cooperation, not only between the next of kin and the health care services but also within and between the different fields of the health care system, make them feel uncertain and insecure in the transition process. The next of kin feel forced to take initiatives to obtain information and responsibility to compensate for inadequacies in the health care system. They appreciate sharing information with professionals in the transition process and perceive that they strive to be involved and collaborate. During the transition process, they experience that their everyday lives are affected in different ways. They have to deal with the frail older person, the disease, and its consequences. Accordingly, they have to relate to different kinds of changes and endeavour to make the best of the situation for the patient in transition.

### Structural analysis

Two themes and four subthemes, closely related to each other, arose from the structural analysis of the text. The themes reflected the meaning of the next of kin’s lived experiences during the patient’s transition process from hospital to home as narrated by them. Though there were unique variations, the following themes and subthemes were valid for the different participants: “Balancing strength and vulnerability,” with the two subthemes “enduring emotional stress” and “striving to maintain security and continuity”; and “Coping with an altered everyday life,” with the two subthemes “dealing with changes” and “being in readiness.”

An overview of the themes and subthemes are presented in Table I. They will be further deepened.

**Table I:** An overview of the themes and subthemes.

Themes	Balancing vulnerability and strength	Coping with an altered everyday life
Subthemes	Enduring emotional stress and frustrations Striving to maintain security and continuity	Dealing with changes Being in readiness

## Balancing vulnerability and strength

This theme reflects the next of kin’s endeavours to deal with more or less unfamiliar health care contexts and worries for a beloved, frail mother, father, or spouse in transition from hospital to home. In these circumstances, the next of kin endure emotional stress and frustrations, and also strive to maintain security and continuity in the older person’s everyday life.

*Enduring emotional stress and frustrations* means dealing with feelings of uncertainty, insecurity, and disappointment. Next of kin expect and want to be included in the discharge process of the older patient. Being excluded from this process is perceived as loosing significant information about the older patient’s state of health and prospects. One next of kin said, “I would just love to be informed about what had happened at the hospital—what they found out and what they decided and what should be taken into account ahead.” Next of kin are also afraid that the patient is discharged too early and are concerned that he/she misunderstands or forgets significant information he/she receives at the hospital. When the transfer time comes as a surprise, it becomes difficult to prepare and organize for the patient’s homecoming. This generates worries and feelings of stress, and loss of control. Furthermore, emotional stress and frustrations are expressed regarding the absence of communication they had hoped for during the patient’s hospital stay. As the staff always seem busy and avoids contact with them, next of kin feel they are invisible and also fail to ask the questions they have.

Enduring emotional stress and frustrations also means feeling uncertain and concerned about the older patient’s illness, symptoms, and condition at the homecoming. This situation makes next of kin tense as they feel they have a lot of responsibility without quite knowing what they are responsible for or how to deal with the situation. In the words of one next of kin,

When the home care nurse came at her first visit and said that she was sorry she had not managed to get the right medicines for my mother, I felt helpless. I wondered what to do and felt at the same time responsible and powerless.

The homecoming is also perceived to be crucial, as next of kin fear what might happen if the older person cannot manage at home. This means that they feel stressed and on alert all the time.

Enduring emotional stress and frustrations also means dealing with failures and misunderstandings in the communication between the hospital and home care, and among the home care professionals. On such occasions, next of kin become insecure and upset, feeling afraid of the consequences for the patient. One next of kin said, “Is it my job to monitor that [the cooperation] works? It is simply unnecessary and frustrating. There are a lot of nice people on each mound, but there is something about the interaction that does not work.”

Their own relationship with the home care nurses is also a centre of attention. Next of kin find the nurses to be caring and doing their best. Still, they see the need for improving communication and cooperation, but find it hard to talk about it as they recognize the overall dependency they are in.

*Striving to maintain security and continuity* contains the next of kin’s perceptions of being monitors in the transition process. This means feeling an overall responsibility for the patient in transition, and it seems to be related to the next of kin’s concerns for the patient, distrust in the health care settings, and never taking anything for granted. They, for example, control the older person’s drugs and medication, sort out various services that are requisitioned for him or her after the hospital stay, and try to ensure that there are no misunderstandings in the communication between or within the hospital and community health care services. One next of kin said, “I can’t trust them, so I just have to check it up. If one only had gotten information from the hospital and felt safe that the communication between the home care and hospital was ok.” Striving to maintain security and continuity means that the next of kin feel they are indispensable in the process, making them more conscious of the patient’s helplessness and dependency. As one next of kin mentioned,

I knew it when the medicine list came, there stands X (name of medication)! Even though I had said “please wipe it out”; he did not tolerate it at the hospital, he won’t tolerate it better at home! He gets sick and vomits! What should he have done without close next of kin! It is frightening—he is unable to remember it!

Narrations that can be associated with a lack of clarity in roles and responsibilities in and between the different health care areas reflect the next of kin’s resignation and sometimes distrust in the situation. One next of kin said,

As soon as you leave the hospital your purchaser is replaced with another. It’s evident that information is not automatically passed on. I had to ask and remind several times and in several places. And God knows how long time this would have taken if I hadn’t nagged.

The narratives also demonstrate that striving to maintain security and continuity means meeting difficulties and challenges but also being proud of one’s own involvement. According to one next of kin,

No one in the system took responsibility for providing the speech therapist he so badly needed. One should be quite resourceful in handling this and to understand, and to orientate oneself in the system. It’s really hard work!

## Coping with an altered everyday life

This theme reflects the emotional, existential, and practical consequences the next of kin face in everyday life when the older person in transition returns from hospital to home. Coping with an everyday life that is altered involves dealing with changes and being in constant readiness.

*Dealing with changes* means addressing changes that affect one’s own as well as the older person’s everyday life. Despite visits from home care personnel, they feel obliged to look after the patient. Thus, they adjust their own daily lives in order to cope with the situation. This means they are flexible and ready to make alterations, and also to forsake their own tasks in everyday life. One next of kin said, “I have to visit my father daily, so I have to adjust my own routines.” Another one mentioned, “Our family had its own shift system during the first period after mum returned home, as she was anxious all the time.”

Stories about the necessity of supporting both the older person and his spouse in the transition process reflect extended responsibilities but also role changes in the family during the transition process. As one next of kin stated, “It’s not only my dad’s role that is changed; I feel I’ve got a new role too, a mummy-role.” Taking responsibility for different kinds of everyday tasks and also for the older person’s spouse in the transition process seems to be perceived as quite natural, although it is talked about as a “double burden.” The old and often frail spouse also has to deal with changes. Feelings of helplessness and unhappiness emerge when being incapable of helping a sick husband or wife. In the words of one spouse, “Sometimes she says ‘my pain kills me’, she vomits and she has pains, and I say to myself, should she be in such pain? Oh, I feel so unhappy, but what can I do?”

Dealing with changes also means having to deal with contradictory feelings. The older person’s continued dependence on home care generates such feelings. On one hand, next of kin feel relieved and grateful for the professional support the older person receives; on the other hand, they feel exposed and frustrated by the situation. The home care nurses are perceived as strangers but at the same time as highly necessary and valued supporters. By their entrance into the family home, next of kin feel that their private matters are disclosed. Furthermore, they feel that the dependency they are in is a reminder that life has changed and is fragile. These feelings seem to be perceived as challenging and influence the next of kin’s sensitivity in the situation. If the home nurse is regarded as emphatic and her activities towards the patient as caring, next of kin appear to find it easier to be familiar and open and to reconcile with the situation, than if the opposite is true. One next of kin told,

It was really hard to realize that he needed home care. I panicked when they said they would come five times a day and at nights too. I thought we would be invaded! But I had no chance to give him the care he needed on my own (…). Although he got a new bed and they came in the middle of the night, I didn’t want to move out from the bedroom we had shared for so many years. But they are so sweet, and I feel so taken care of. They really care for me too!

Dealing with changes is also shown in next of kin’s stories related to changes in the patient’s appearances, in the physical as well as mental state, and in the environment as, for example, in association with new aids and technical equipment. The stories reflect that dealing with the changes means moving between accepting and not accepting the older person’s state of health and life situation; of feeling despair as well as hope. Acceptance means realizing the irreversibility of the situation, cooperating with home care, and making the best of it. As one next of kin mentioned,

And we, the family, have also, if I may say so, tried to do our best to both understand how this apparatus work and to lessen the fear of it. And we have also tested out ways of reducing the problems this treatment causes for our mum. Thus, we have given [the home care] some input, since I think or I guess they haven’t faced a situation like this very often.

Dealing with changes means feeling that the health care system is or is not taking one’s situation seriously. Becoming conscious of and recognizing significant changes and helplessness in the older person’s situation generates feelings of sadness but also of responsibility among the next of kin. In their efforts to help the older person, they interact with health care professionals, and perceive the interactions as both positive and negative. Positive interactions mean that that next of kin feel accommodated, and that the situation as such is understood. Negative interactions mean that next of kin feel disappointed, resigned, angry, and let down. One next of kin told,

He used to be a positive and engaged man. Now he seems confused and depressed. I felt I had to help him after he came home. So I arranged for a meeting with the community services and asked if he had to get worse to get further help. I could see that my question trigged her. And she answered, “there are many who have it worse than your father waiting for a place in the nursing home.” I was so desperate!

*Being in readiness* means being available and ready to act at any time when the older person returns from hospital to home, and this has a lot in common with the former subtheme. Next of kin perceive that the older patient is discharged too early and that the home nurses are too busy and relate this to the necessity of being in readiness. Being in readiness means putting one’s own needs aside to stand up for the older person whenever required. Such needs might mean turning down social activities or refraining from drinking alcohol in parties just in case they are called on. One next of kin said, “It’s like being on your toes, waiting for a phone call and I have my mobile at my bedside at all times.” According to their stories, next of kin, mainly sons and daughters, seem to be available for the older person or his or her spouse 24 h a day in the initial time home. In the words of one next of kin, “We are very lucky as we have only 3 min to drive to my parents’ house.”

Although being in readiness seems to be perceived as an obvious priority, next of kin’s stories reflect feelings of being exhausted, mentally challenged, and constrained in the situation.

### Comprehensive understanding

The next of kin’s experiences of the frail older person’s transition from hospital to home are characterized by movements between human vulnerability and human agency. Uncertainty related to the older person’s illness, and how the present situation will influence the older person’s close and more distant future fills them with insecurity and worries. In these circumstances they are sensing their own, as well as the older person’s vulnerability, and thus are facing life’s fragility. In this unfamiliar situation, they feel significant for the frail older person but insignificant for the health care providers. Balancing these feelings not only generates stress but also strength and vigour within the next of kin. They are called to hold continuity in the life of the older person and hence adjust their own, and assist the professional care to maintain human dignity in the transition process.

## Discussion

Next of kin face several challenges during the transition of an older patient from hospital to home. They have to adapt to a complex situation in which they are vulnerable human beings as well as significant agents. In the present study, the next of kin reported that they were frustrated over not taking part in the planning of the discharge from the hospital. They also described lack of involvement, communication, and information in the transition process. This made them insecure and caused emotional stress. Several studies show that next of kin play a major and significant role in supporting older persons during hospitalization and especially after discharge (Dale, Sævareide, Kirkevold, & Soderhamn, 2008; Naylor & Keating, 2008). Consequently, they are also affected by the older person’s situation and thus want to be involved and participate in the discharge process (Bragstad, Kirkevold, Hofoss, & Foss, 2014). Such participation gives them the opportunity to influence as well as to be informed about the decisions that are made. Next of kin in this study wanted to be involved in the discharge process to be able to arrange and be prepared for the patient’s homecoming. They were also concerned that the older person would misunderstand information that was given about the health condition. Therefore, they wanted to receive the same information to be better prepared to support the older person after discharge. Substantial unmet needs regarding information and lack of family involvement in the transition process have been described in several studies over years (Dedhia et al., 2009; Dunnion & Kelly, 2008; Golden et al., 2010; Naylor, 2002). Previous studies as well as the current study thus demonstrate that the Norwegian government’s ambition of improving user involvement is essential to focus on when frail older persons are transferred from hospital to home (Norwegian Ministry of Health and Care Services, Report No. 10, 29, 47). This requires that health care professionals also focus on the next of kin and attend to their needs during the transition process.

The goal of improving user involvement and thereby empowering the patient as well as the next of kin can be considered a move towards relation-centred care as described by Nolan et al. (2004) or person-centred care as described by McCance, McCormack, and Dewing (2012). Both approaches are embedded in ideals encompassing the development of a relationship built on respect, mutual trust, and understanding. This increasing emphasis on user involvement in health and social care is, in Galvin and Todres’ opinion (2013), a reaction to the traditional medical model which has emphasized a view of the person as passively subjected to internal and external forces. This means that the person is rendered passive in relation to their condition and treatment. Next of kin in this study described feelings of being invisible and overlooked by the health care providers, and a health care system characterized by efficiency with little attention and time for communication. Attitudes and practices that provoke such feelings may harm human dignity and thus have the potential for dehumanization. Thus, health care professionals need to be aware of how lack of attention can make next of kin feel undervalued as respected agents and persons. Conversely, care can be humanized by enhancing agency through increased patient participation (Galvin & Todres, 2013). According to McCormack and McCance (2010), the knowledge and experience that each person brings to the care situation is crucial for user involvement and shared decision making. Narratives in our study reflect absence of shared knowledge and information. This absence may have deprived next of kin control in the transition process. The uncertainty they felt, their concerns for the patient, and distrust in the health care settings can be understood in this perspective.

Frail older persons are often exposed to avoidable transitions between various sectors in the health care system. With vastly different goals and few bridges to connect the sectors, these transitions are exposed to serious breakdowns in care (Naylor, 2012). Experiencing deficiencies in the information that was exchanged between the hospital and home care nurses, next of kin feared the consequences and took responsibility to sort out and compensate for the failures. In this way they worked as bridges between the hospital and the community health care. However, this was an unintended role that made them insecure and frustrated. It points to the significance of persisting efforts to exchange accurate and complete information across health care settings (Naylor, 2006). Next of kin reported a high number of home care nurses in the initial time after discharge and perceived this as a threat to the quality of care. Nolan et al. claim that in the context of a caring relationship next of kin need to be ensured that competent standards of care are provided, and that the patient’s dignity and integrity, well-being, and personhood are maintained (Nolan et al., 2008). Our findings indicate that next of kin were uncertain whether the older person received care that maintained well-being and safety in the transition process, and thus felt it necessary to observe and assist the professional care. Continuity of care is important for establishing positive relationships (Naylor, 2006). Consistent access to the same professionals after discharge is therefore essential. Continuity of care enables the health care professional to better monitor the situation and to establish a relationship with both the patient and the next of kin. This makes continuity of care fundamental to provide care informed by insight in the other’s experiences and needs. That is lifeworld insights, or insight based on the complex experiences of lives lived as a whole (Galvin & Todres, 2013).

In our narratives it became clear that next of kin struggled with their own emotions connected to the altered situation and simultaneously endeavoured to be supportive agents for the patient in transition. Facing physical and mental changes in the older person and in the relationship with them, together with worries for the present situation and the future evoked feelings of sadness and also of insufficiency. At the same time, their willingness to take responsibility, to be flexible, and to deal with the changed situation was prominent in their stories. Next of kin’s devotions and profound feelings of responsibility for the older person in transition are also reported in a study by Eika et al. (2013). Even if the older person had been moved from home to a nursing home, they kept on feeling responsible and kept an eye on things and also supported their older relative in circumstances that threatened their dignity. This demonstrates how exposed the older person in transition is perceived by the next of kin, but it also reflects their own vulnerability. To avoid neglecting vulnerability, health care professionals must be aware of the meaning of the transition for those who are involved. This is essential for understanding their experiences of it (Schumacher & Meleis, 1994). Meaning is embedded in the person’s lifeworld and must be understood in the context of “a before” and “a next” (Galvin & Todres, 2013). To understand next of kin’s vulnerability, attention to how the changes affect everyday life is demanded. Furthermore, attention must be paid to how the changes influence previous expectations and plans, and what is possible now and in the future.

Policy documents (Report No. 47 [2008–2009] to the Storting, Report No. 29 [2012–2013], and Report No. 10 [2012–2013]) address the need to humanize care. However, an increasing focus on efficiency and cost containment in the prevailing caring practices seems to be a serious threat to this ambition (Bauer et al., 2009; Hvalvik & Dale, 2013; Vabø, 2012). A humanized practice means to see the patient (or the next of kin) in both their agency and vulnerability (Galvin & Todres, 2013). This is in accordance with the next of kin’s wishes in our study. They strongly wanted to be involved and share information in the transition process, and although their vulnerability was less obvious, their desire to be seen as human beings was inherent in their stories. It is crucial that health care professionals acknowledge the humanizing dimension when they consider the complexity of next of kin’s lived situation.

### Strengths and limitations of the study

A phenomenological hermeneutic approach was considered relevant as the aim of this study was to illuminate and gain insight in next of kin’s experiences. Narrative interviews were conducted. This is an appropriate method for disclosing the meaning of lived experience (Lindseth & Norberg, 2004). Those who chose to participate in the study were resourceful and had strong meanings about the transition process; they were mainly daughters. Overall, this may be considered a limitation of the present study. However, their ability to express and describe their experiences resulted in rich amounts of data, which may be regarded as a strength. During the whole research process, the two researchers tried to be conscious of and challenge their pre-understanding. In order to arrive at possible interpretations, rigour was endeavoured throughout the process. A text has never only one meaning (Ricoeur, 1976). The comprehensive understanding of the present study is therefore only one of many possible interpretations. It is, however, the one that the two researchers found most probable and meaningful.

## Conclusion

Our findings suggest that lack of involvement in the transition process strongly influenced next of kin’s lived experiences and made them vulnerable. Incomplete communication among health care providers and across the health care sectors during the transition made the next of kin uncertain and worried. However, in striving to maintain continuity and safety in the older person’s life, they demonstrated strength and were significant agents in the transition process. Health care professionals must see and respect the next of kin as individuals, and involve them in a proper way. This means to accommodate both their vulnerability and their abilities to act, and thereby make them feel valued as respected agents and human beings in the transition process.

## Authors’ contributions

First author designed the study. Both authors collected the data. First author performed the structural analysis and drafted the manuscript. Second author contributed to the interpretation of the findings and critical review of the manuscript. Both authors read and approved the final manuscript.

## Note

1. In Norway most home care services are organized in line with the purchaser–provider model, which is inspired by the New Public Management movement. The purchaser unit is a separate unit in the municipality health care services, where the responsibility for needs assessment is placed. The responsibility for exercising the care and re-examining the care needs is however placed in home care (Vabø, 2012).
